# A novel target convergence set based random walk with restart for prediction of potential LncRNA-disease associations

**DOI:** 10.1186/s12859-019-3216-4

**Published:** 2019-12-03

**Authors:** Jiechen Li, Xueyong Li, Xiang Feng, Bing Wang, Bihai Zhao, Lei Wang

**Affiliations:** 1 College of Computer Engineering & Applied Mathematics, Changsha University, Changsha, Hunan People’s Republic of China; 2 Key Laboratory of Hunan Province for Internet of Things and Information Security, Xiangtan University, XiangTan, People’s Republic of China; 3 School of Electrical and Information Engineering, Anhui University of Technology, Anhui 243002 Maanshan, People’s Republic of China

**Keywords:** Potential lncRNA-disease association prediction, Heterogeneous network, Random walk with restart, Target convergence set, Global set

## Abstract

**Background:**

In recent years, lncRNAs (long-non-coding RNAs) have been proved to be closely related to the occurrence and development of many serious diseases that are seriously harmful to human health. However, most of the lncRNA-disease associations have not been found yet due to high costs and time complexity of traditional bio-experiments. Hence, it is quite urgent and necessary to establish efficient and reasonable computational models to predict potential associations between lncRNAs and diseases.

**Results:**

In this manuscript, a novel prediction model called TCSRWRLD is proposed to predict potential lncRNA-disease associations based on improved random walk with restart. In TCSRWRLD, a heterogeneous lncRNA-disease network is constructed first by combining the integrated similarity of lncRNAs and the integrated similarity of diseases. And then, for each lncRNA/disease node in the newly constructed heterogeneous lncRNA-disease network, it will establish a node set called TCS (Target Convergence Set) consisting of top 100 disease/lncRNA nodes with minimum average network distances to these disease/lncRNA nodes having known associations with itself. Finally, an improved random walk with restart is implemented on the heterogeneous lncRNA-disease network to infer potential lncRNA-disease associations. The major contribution of this manuscript lies in the introduction of the concept of TCS, based on which, the velocity of convergence of TCSRWRLD can be quicken effectively, since the walker can stop its random walk while the walking probability vectors obtained by it at the nodes in TCS instead of all nodes in the whole network have reached stable state. And Simulation results show that TCSRWRLD can achieve a reliable AUC of 0.8712 in the Leave-One-Out Cross Validation (LOOCV), which outperforms previous state-of-the-art results apparently. Moreover, case studies of lung cancer and leukemia demonstrate the satisfactory prediction performance of TCSRWRLD as well.

**Conclusions:**

Both comparative results and case studies have demonstrated that TCSRWRLD can achieve excellent performances in prediction of potential lncRNA-disease associations, which imply as well that TCSRWRLD may be a good addition to the research of bioinformatics in the future.

## Background

For many years, the genetic information of organism is considered to be stored only in genes used for protein coding, and RNAs have always been thought to be an intermediary in the process of encoding proteins by DNAs [1, 2]. However, recent studies have shown that the genes used to encode proteins only account for a small part (less than 2%) of human genome and more than 98% of human genome are not made up of genes that encode proteins and yield a big mount of ncRNAs (non-coding-RNAs) [3, 4]. In addition, as the complexity of biological organisms increases, so does the importance of ncRNAs in biological processes [5, 6]. Generally, ncRNAs can be divided into two major categories such as small ncRNAs and long ncRNAs (lncRNAs) according to the length of nucleotides during transcription, where small ncRNAs consist of less than 200 nucleotides and include microRNAs and transfer RNAs etc. However, lncRNAs consist of more than 200 nucleotides [7–9]. In 1990, the first two kinds of lncRNAs such as H19 and Xist were discovered by researchers through gene mapping. Since gene mapping approach is extremely time-consuming and labor-intensive, then researches in the field of lncRNAs have been at a relatively slow pace for a long time [10, 11]. In recent years, with the rapid development of high-throughput technologies in gene sequencing, more and more lncRNAs have been found in eukaryotes and other species [12, 13]. Moreover, simulation results have shown as well that lncRNAs play important roles in various physiological processes such as cell differentiation and death, regulation of epigenetic shape and so on [8, 14, 15]. Simultaneously, growing evidences have further illustrated that lncRNAs are closely linked to diseases that pose a serious threat to human health [16–18], which means that lncRNAs can be used as potential biomarkers in the course of disease treatment in the future [19].

With the discovery of a large number of new types of lncRNAs, many databases related to lncRNAs such as lncRNAdisease [20], lncRNAdb [21], NONCODE [22] and Lnc2Cancer [23] have been established by researchers successively, however, in these databases, the number of known associations between lncRNAs and diseases is still very limited due to high costs and time-consumption of traditional biological experiments. Thus, it is meaningful to develop mathematical models to predict potential lncRNA-disease associations quickly and massively. Based on the assumption that similar diseases tend to be more likely associated with similar lncRNAs [24, 25], up to now, a good deal of computational models for inferring potential lncRNA-disease associations have been proposed. For instance, Chen et al. proposed a computational model called LRLSLDA [26] for prediction of potential lncRNA-disease associations by adopting the method of Laplacian regularized least squares. Ping and Wang et al. constructed a prediction model for extracting feature information from bipartite interactive networks [27]. Zhao and Wang et al. developed a computational model based on Distance Correlation Set to uncover potential lncRNA-disease associations through integrating known associations between three kinds of nodes such as disease nodes, miRNA nodes and lncRNA nodes into a complex network [28]. Chen et al. proposed an lncRNA-disease association prediction model based on a heterogeneous network by considering the influence of path length between nodes on the similarity of nodes in the heterogeneous network [29–31]. However, for some time past, a network traversal method called RWR (Random Walk with Restart) has emerged in the field of computational biology including prediction of potential miRNA-disease associations [32, 33], drug-target associations [34] and lncRNA-disease associations [35–37] etc.

Inspired by the thoughts illustrated in above state-of-the-art literatures, in this paper, a computational model called TCSRWRLD is proposed to discover potential lncRNA-disease associations. In TCSRWRLD, a heterogeneous network is constructed first through combining known lncRNA-disease associations with the lncRNA integrated similarity and the disease integrated similarity, which can overcome a drawback of traditional RWR based approaches that these approaches cannot start walking process while there are no known lncRNA-disease associations. And then, each node in the heterogeneous network will establish its own TCS according to the information of network distance, which can reflect the specificity of different nodes in the walking process and make the prediction more accurate and less time-consuming. Moreover, considering that for a given walker, while its TCS has reached the ultimate convergence state, there may be still some nodes that are not included in its TCS but actually associated with it, then in order to ensure that there is no omission in our prediction results, each node in the heterogeneous network will further establish its own GS as well. Finally, for evaluating the prediction performance of our newly proposed model TCSRWRLD, cross validation are implemented based on known lncRNA-disease associations downloaded from the lncRNAdisease database (2017version), and as a result, TCSRWRLD can achieve reliable AUCs of 0.8323, 0.8597, 0.8665 and 0.8712 under the frameworks of 2-folds CV, 5-folds CV, 10-folds CV and LOOCV respectively. In addition, simulation results in case studies of leukemia and lung cancer show that there are 5 and 7 out of the top 10 predicted lncRNAs having been confirmed to be associated with Leukemia and Lung cancer respectively by recent evidences, which demonstrate as well that our model TCSRWRLD has excellent prediction performance.

## Results

In order to verify the performance of TCSRWRLD in predicting potential lncRNA-disease associations, LOOCV, 2-folds CV, 5-folds CV and 10-folds CV were implemented on TCSRWRLD respectively. And then, based on the dataset of 2017-version downloaded from the lncRNADisease database, we obtained the Precision-Recall curve (P-R curve) of TCSRWRLD. In addition, based on the dataset of 2017-version downloaded from the lncRNADisease database and the dataset of 2016-version downloaded from the lnc2Cancer database, we compared TCSRWRLD with state-of-the-art prediction models such as KATZLDA, PMFILDA [38] and Ping’s model separately. After that, we further analyzed the influences of key parameters on the prediction performance of TCSRWRLD. Finally, case studies of leukemia and lung cancer were performed to validate the feasibility of TCSRWRLD as well.

### Cross validation

**Fig. 1:**
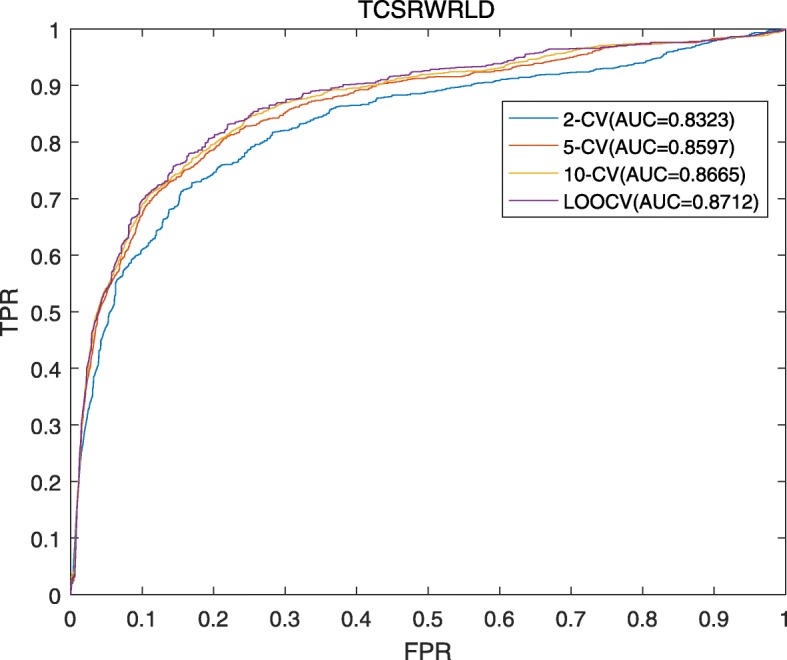
AUCs achieved by TCSRWRLD under the frameworks of 2-folds CV, 5-folds CV, 10-folds CV and LOOCV respectively

In this section, ROC curve (Receiver Operating Characteristic) and the score of AUC (Area Under ROC Curve) will be adopted to measure the performance of TCSRWRLD in different cross validations. Here, let TPR (True Positive Rates or Sensitivity) represent the percentage of candidate lncRNAs-disease associations with scores higher than a given score cutoff, and FPR (False Positive Rates or 1-Specificity) denote the ratio of predicted lncRNA-disease associations with scores below the given threshold, then ROC curves can be obtained by connecting the corresponding pairs of TPR and FPR on the graph. As illustrated in Fig. 1, simulation results show that TCSRWRLD can achieve reliable AUCs of 0.8323, 0.8597, 0.8665 and 0.8712 in the frameworks of 2-folds CV, 5-folds CV, 10-folds and LOOCV respectively, which implies that TCSRWRLD can achieve excellent performance in predicting potential lncRNA-disease associations.

**Fig. 2:**
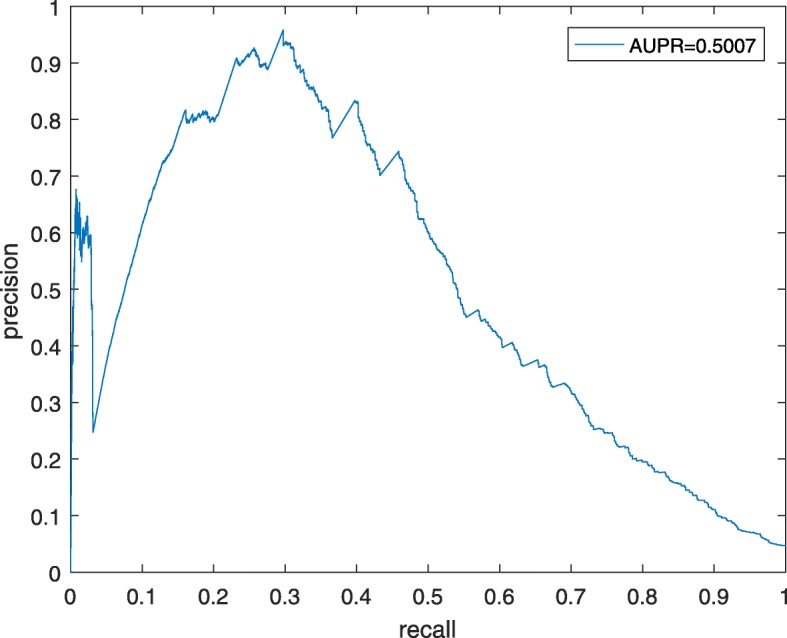
precision-recall curve achieved by TCSRWRLD

Moreover, in order to further estimate the prediction performance of TCSRWRLD, we will obtain the P-R curve of TCSRWRLD as well. Unlike the AUC, the AUPR (Area Under the Precision-Recall curve) represents the ratio of all true positives to all positive predictions at every given recall rate. As illustrated in Fig. 2, simulation results show that TCSRWRLD can achieve a reliable AUPR of 0.5007.

### Comparison with other related methods

**Fig. 3:**
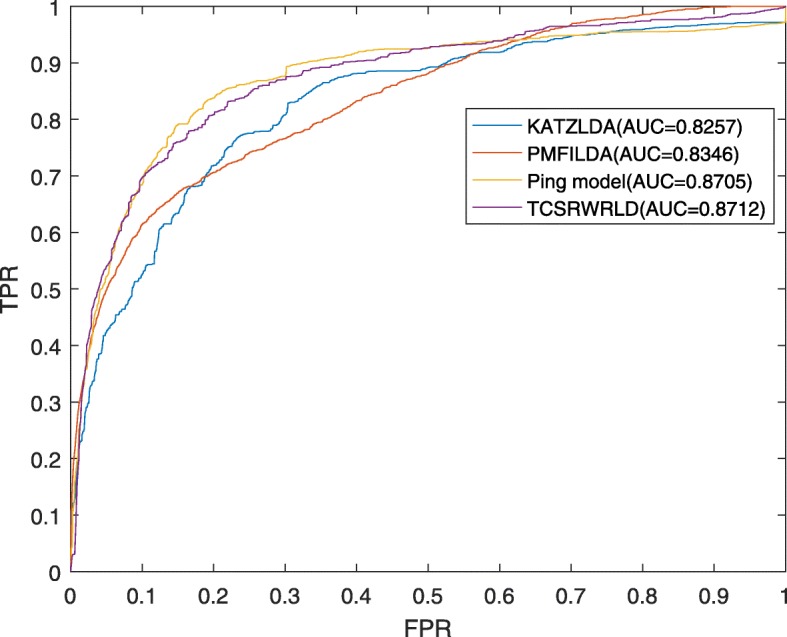
The AUCs achieved by TCSRWRLD, KATZLDA, Ping’s model and PMFILDA in LOOCV based on the dataset of 2017-version downloaded from the lncRNAdisease database

From above descriptions, it is easy to know that TCSRWRLD can achieve satisfactory prediction performance. In this section, we will compare TCSRWRLD with some classical prediction models to further demonstrate the performance of TCSRWRLD. Firstly, based on the dataset of 2017-version downloaded from the lncRNAdisease database, we will compare TCSRWRLD with the state-of-the-art models such as KATZLDA, PMFILDA and Ping’s model. As shown in Fig. 3, it is easy to see that TCSRWRLD can achieve a reliable AUC of 0.8712 in LOOCV, which is superior to the AUCs of 0.8257, 0.8702 and 0.8346 achieved by KATZLDA, Ping’s model and PMFILDA in LOOCV respectively.

**Fig. 4:**
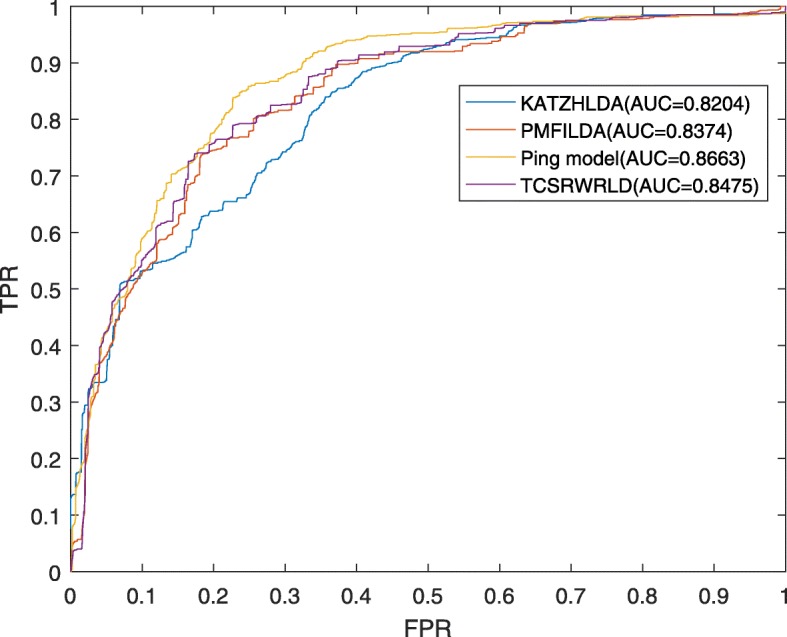
the AUCs achieved by TCSRWRLD, KATZLDA, Ping’s model and PMFILDA based on the dataset of 2016-version downloaded from the Lnc2Cancer database

Moreover, in order to prove that TCSRWRLD can perform well in different data backgrounds, we also adopt the dataset of 2016-version downloaded from the lnc2Cancer database, which consists of 98 human cancers, 668 lncRNAs and 1103 confirmed associations between them, to compare TCSRWRLD with KATZLDA, PMFILDA and Ping’s model. As illustrated in Fig. 4, it is easy to see that TCSRWRLD can achieve a reliable AUC of 0.8475 in LOOCV, which is superior to the AUCs of 0.8204 and 0.8374 achieved by KATZLDA and PMFILDA respectively, while is inferior to the AUC of 0.8663 achieved by Ping’s model.

### Analysis on effects of parameters

In TCSRWRLD, there are some key parameters such as ${\gamma}_l^{\prime }$, ${\gamma}_d^{\prime }$ and *∂*. As for ${\gamma}_l^{\prime }$ and ${\gamma}_d^{\prime }$ in the Equation (5) and Equation (11), we have already known that the model can achieve the best performance when the values of ${\gamma}_l^{\prime }$and${\gamma}_d^{\prime }$ are both set to 1 [39]. Hence, in order to estimate effect of the key parameter *∂* on the prediction performance of TCSRWRLD, we will set the value range of *∂* from 0.1 to 0.9 and select the value of AUC in LOOCV as the basis of parameter selection in this section. As illustrated in Table 1, It is easy to see that TCSRWRLD can achieve the highest value of AUC in LOOCV while *∂* is set to 0.4. Moreover, it is also easy to see that TCSRWRLD can maintain robustness for different values of *∂*, which means that TCSRWRLD is not sensitive to the values of *∂* as well.

**Table 1:** AUCs achieved by TCSRWRLD in LOOCV while the parameter *∂* is set to different values from 0.1 to 0.9

*∂*	0.1	0.2	0.3	0.4	0.5	0.6	0.7	0.8	0.9
AUC	0.8418	0.8655	0.8707	0.8712	0.8700	0.8680	0.8700	0.8672	0.8660

### Case studies

Up to now, cancer is considered as one of the most dangerous diseases to human health because it is hard to be treated [40]. At present, the incidence of various cancers has a high level not only in the developing countries where medical development is relatively backward, but also in the developed countries where the medical level is already very high. Hence, in order to further evaluate the performance of TCSRWRLD, case study of two kinds of dangerous cancers such as lung cancer and leukemia will be implemented in this section. As for these two kinds of dangerous cancers, the incidence of lung cancer has remained high in recent years, and the number of lung cancer deaths per year is about 1.8 million, which is the highest of any cancer types. However, the survival rate within five years after the diagnosis of lung cancer is only about 15%, which is much lower than that of other cancers [41]. Recently, growing evidences have shown that lncRNAs play crucial roles in the development and occurrence of lung cancer [42]. As illustrated in Table 2, while implementing TCSRWRLD to predict lung cancer related lncRNAs, there are 7 out of the top 10 predicted candidate lung cancer related lncRNAs having been confirmed by the latest experimental evidences. Additionally, as a blood-related cancer [43], Leukemia has also been found to be closely related to a variety of lncRNAs in recent years. As illustrated in Table 2, while implementing TCSRWRLD to predict Leukemia related lncRNAs, there are 5 out of the top 10 predicted candidate Leukemia related lncRNAs having been confirmed by state-of-the-art experiment results as well. Thus, from above simulation results of case studies, we can easily reach an agreement that TCSRWRLD may have great value in predicting potential lncRNA-disease associations.

**Table 2:** Evidences of top 10 potential leukemia-related lncRNAs and lung cancer-related lncRNAs predicted by TCSRWRLD

Disease name	LncRNA name	RANK	Evidence
Lung cancer	YUG1	1	Lnc2Cancer
Lung cancer	XIST	2	Lnc2Cancer
Lung cancer	PVT1	4	Lnc2Cancer
Lung cancer	PCAT29	5	MNDR
Lung cancer	HOTAIRM1	7	MNDR
Lung cancer	NEAT1	9	Lnc2Cancer
Lung cancer	anti-NOS2A	10	Lnc2Cancer
Leukemia	MALAT1	1	Lnc2Cancer
Leukemia	HOTAIR	2	Lnc2Cancer
Leukemia	H19	4	Lnc2Cancer
Leukemia	MEG3	5	Lnc2Cancer
Leukemia	PVT1	7	Lnc2Cancer

## Discussion

Since it is very time-consuming and labor-intensive to verify associations between lncRNAs and diseases through traditional biological experiments, then it has become a hot topic in bioinformatics to establish computational models to infer potential lncRNA-disease associations, which can help researchers to have a deeper understanding of diseases at the lncRNA level. In this manuscript, a novel prediction model called TCSRWRLD is proposed, in which, a heterogeneous network is constructed first through combining the disease integrated similarity, the lncRNA integrated similarity and known lncRNA-disease associations, which can guarantee that TCSRWRLD is able to overcome the shortcomings of traditional RWR based prediction models that the random walk process cannot be started while there are no known lncRNA-disease associations. And then, based on the newly constructed heterogeneous network, a random walk based prediction model is further designed based on the concepts of TCS and GS. In addition, based on the dataset of 2017-version downloaded from the lncRNAdisease database, a variety of simulations have been implemented, and simulation results show that TCSRWRLD can achieve reliable AUCs of 0.8323, 0.8597 0.8665 and 0.8712 under the frameworks of 2-fold CV, 5-fold CV, 10-fold CV and LOOCV respectively. Additionally, simulation results of case studies of lung cancer and leukemia show as well that TCSRWRLD has a reliable diagnostic ability in predicting potential lncRNA-disease associations. Certainly, the current version of TCSRWRLD still has some shortages and deficiencies. For example, the prediction performance of TCSRWRLD can be further improved if more known lncRNA-disease associations have been added into the experimental datasets. In addition, more accurate establishment of Mesh database will help us obtain more accurate disease semantic similarity scores, which is very important for the calculation of lncRNA functional similarity as well. Of course, all these above problems will be the focus of our future researches.

## Conclusion

In this paper, the main contributions are as follows: (1) A heterogeneous lncRNA-disease network is constructed by integrating three kinds of networks such as the known lncRNA-disease association network, the disease-disease similarity network and the lncRNA-lncRNA similarity network. (2) Based on the newly constructed heterogeneous lncRNA-disease network, the concept of network distance is introduced to establish the TCS (Target Convergence Set) and GS (Global Set) for each node in the heterogeneous lncRNA-disease network. (3) Based on the concepts of TCS and GS, a novel random walk model is proposed to infer potential lncRNA-disease associations. (4) Through comparison with traditional state-of-the-art prediction models and the simulation results of case studies, TCSRWRLD is demonstrated to be of excellent prediction performance in uncovering potential lncRNA-disease associations.

## Methods and materials

### Known disease-lncRNA associations

Firstly, we download the 2017-version of known lncRNA-disease associations from the lncRNAdisease database (http://www.cuilab.cn/ lncrnadisease). And then, after removing duplicated associations and picking out the lncRNA-disease associations from the raw data, we finally obtain 1695 known lncRNA-disease associations (see Additional file 1) including 828 different lncRNAs (see Additional file 2) and 314 different diseases (see Additional file 3). Hence, we can construct a 314 × 828 dimensional lncRNA-disease association adjacency matrix *A*, in which, there is *A*(*i*, *j*) = 1, if and only if there is an known association between the disease *d*_*i*_ and the lncRNA *l*_*j*_ in the LncRNADisease database, otherwise there is *A*(*i*, *j*) = 0. In addition, for convenience of description, let *N*_*L*_ = 828 and *N*_*D*_ = 314, then it is obvious that the dimension of the lncRNA-disease association adjacency matrix *A* can be represented as *N*_*D*_ × *N*_*L*_. And the like mentioned above, we can get a cancer-disease associations adjacency matrix which dimension is 98 × 668 (It comes from 2016-version of known lncRNA-disease associations from the Lnc2Cancer database) (see Additional file 4).

### Similarity of diseases

#### Semantic similarity of diseases

In order to estimate the semantic similarity between different diseases, based on the concept of DAGs (Directed Acyclic Graph) of different diseases proposed by Wang et al. [44, 45], we can calculate the disease semantic similarity through calculating the similarity between compositions of DAGs of different diseases as follows:

##### Step 1

**Fig. 5:**
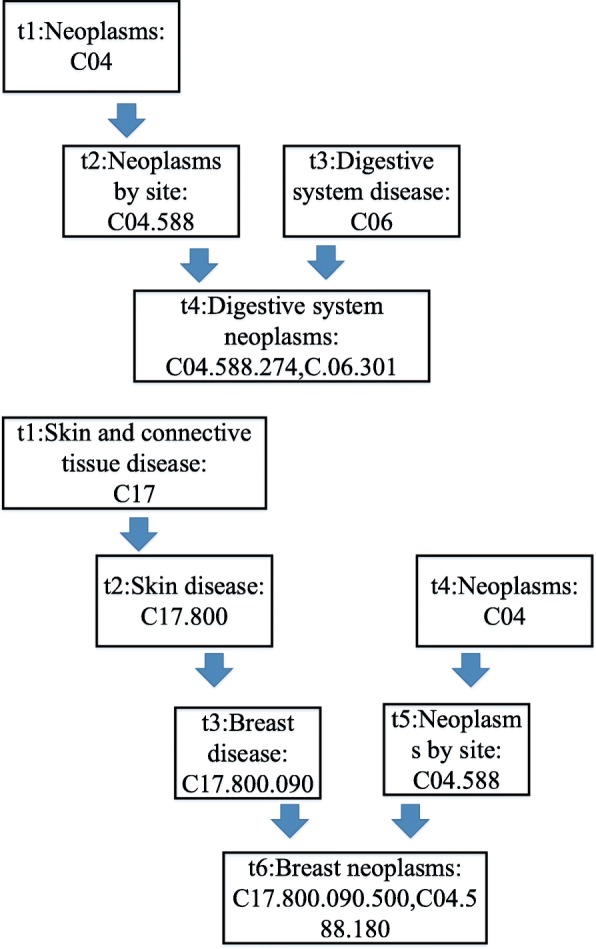
DAG of the digestive system neoplasms and breast neoplasms

For all these 314 diseases newly obtained from the lncRNAdisease database, their corresponding MESH descriptors can be downloaded from the Mesh database in the National Library of Medicine (http://www.nlm.nih.gov/). As illustrated in Fig. 5, based on the information of MESH descriptors, each disease can establish a DAG of its own.

##### Step 2

For any given disease *d*, Let its DAG be DAG(*d*) = (*d*, *D*(*d*), *E*(*d*)), where *D*(*d*) represents a set of nodes consisting of the disease *d* itself and its ancestral disease nodes, and *E*(*d*) denotes a set of directed edges pointing from ancestral nodes to descendant nodes.

##### Step 3

For any given disease *d* and one of its ancestor nodes *t* in DAG(*d*), the semantic contributions of the ancestor node *t* to the disease *d* can be defined as follows:

1 $$ {D}_d(t)=\left\{\begin{array}{c}1\\ {}\max \left\{\varDelta \ast {D}_d\left(t\hbox{'}\right)|t\hbox{'}\in children\kern0.17em of\;t\right\}\kern1em \begin{array}{c} if\;t=d\\ {} if\;t\ne d\end{array}\end{array}\right\} $$

Where Δ is the attenuation factor with value between 0 and 1 to calculate the disease semantic contribution, and according to the state-of-the-art experimental results, the most appropriate value for*Δ*is 0.5 .

##### Step 4

For any given disease *d*, let its DAG be DAG(*d*), then based on the concept of DAG, the semantic value of *d* can be defined as follows:

2 $$ D(d)={\sum \limits}_{t_i\in DAG(d)}{D}_d\left({t}_i\right) $$

Taking the disease DSN (Digestive Systems Neoplasms) illustrated in Fig. 5 for example, according to the Equation (1), it is easy to know that the semantic contribution of digestive systems neoplasms to itself is 1. Besides, since the neoplasms by site and the digestive system disease located in the second layer of the DAG of DSN, then it is obvious that both of the semantic contributions of these two kinds of diseases to DSN are 0.5*1 = 0.5. Moreover, since the neoplasms located in the third layer of the DAG of DSN, then its semantic contribution to DSN is 0.5*0.5 = 0.25. Hence, according to above formula (2), it is easy to know the semantic value of DSN will be 2.25 (=1 + 0.5 + 0.5 + 0.25).

##### Step 5

For any two given diseases *d*_*i*_ and *d*_*j*_, based on the assumption that the more similar the structures of their DAGs, the higher the semantic similarity between them will be, the semantic similarity between *d*_*i*_ and *d*_*j*_ can be defined as follows:

3 $$ DisSemSim\left(i,j\right)= DisSemSim\left({d}_i,{d}_j\right)=\frac{\sum_{t\in \left( DAG\left({d}_i\right)\cap DAG\left({d}_j\right)\right)}\left({D}_{d_i}(t)+{D}_{d_j}(t)\right)}{D\left({d}_i\right)+D\left({d}_j\right)} $$

### Gaussian interaction profile kernel similarity of diseases

Based on the assumption that similar diseases tend to be more likely associated with similar lncRNAs, according to above newly constructed lncRNA-disease association adjacency matrix *A*, for any two given diseases *d*_*i*_ and *d*_*j*_, the Gaussian interaction profile kernel similarity between them can be obtained as follows:

4 $$ GKD\left({d}_i,{d}_j\right)=\mathit{\exp}\left(-{\gamma}_d{\left\Vert IP\left({d}_i\right)- IP\left({d}_j\right)\right\Vert}^2\right) $$

5 $$ {\gamma}_d={\gamma}_d^{\hbox{'}}/\left({\sum \limits}_{k=1}^{N_D}{\left\Vert IP\left({d}_k\right)\right\Vert}^2\right) $$

Here, *IP*(*d*_*t*_) denotes the vector consisting of elements in the *t*th row of the lncRNA-disease adjacency matrix *A*. *γ*_*d*_ is the parameter to control the kernel bandwidth based on the new bandwidth parameter ${\gamma}_d^{\prime }$by computing the average number of lncRNAs-disease associations for all the diseases. In addition, inspired by the thoughts of former methods proposed by O. Vanunu et al. [46], we will adopt a logistics function to optimize the Gaussian interaction profile kernel similarity between diseases, and based on above Equation (4), we can further obtain a *N*_*D*_ × *N*_*D*_ dimensional adjacency matrix *FKD* as follows:

6 $$ FKD\left(i,j\right)=\frac{1}{1+{e}^{\left(-12 GKD\left(i,j\right)+\log (9999)\right)}} $$

### Integrated similarity of diseases

Based on the disease semantic similarity and disease Gaussian interaction profile kernel similarity obtained above, a *N*_*D*_ × *N*_*D*_ dimensional integrated disease similarity adjacency matrix *KD* (*N*_*D*_ × *N*_*D*_) can be obtained as follows:

7 $$ KD\left(i,j\right)=\frac{DisSemSim\left(i,j\right)+ FKD\left(i,j\right)}{2} $$

### Similarity of LncRNAs

#### Functional similarity of LncRNAs

We can obtain corresponding disease groups of two given lncRNAs *l*_*i*_ and *l*_*j*_ from the known associations of lncRNA-disease. Based on the assumption that similar diseases tend to be more likely associated with similar lncRNAs, We define the functional similarity of two given lncRNAs *l*_*i*_ and *l*_*j*_ as the semantic similarity between the disease groups corresponding to them. The specific calculation process is as follows:

For any two given lncRNAs *l*_*i*_ and *l*_*j*_, let *DS*(*i*) = {*d*_*k*_ | *A*(*k*, *i*) = 1, *k*∈[1, *N*_*D*_]} and *DS*(*j*) = {*d*_*k*_ | *A*(*k*, *j*) = 1, *k*∈[1, *N*_*D*_]}, then the functional similarity between *l*_*i*_ and *l*_*j*_ can be calculated according to the following steps [31]:

##### Step 1

For any given disease group *DS*(*k*) and disease *d*_*t*_∉*DS*(*k*), we first calculate the similarity between *d*_*t*_ and *DS*(*k*) as follows:

8 $$ S\left({d}_t, DS(k)\right)={\max}_{d_s\in DS(k)}\left\{ DisSemSim\left({d}_t,{d}_s\right)\right\} $$

##### Step 2

Therefore, based on above Equation (8), we define the functional similarity between *l*_*i*_ and *l*_*j*_ as *FuncKL*(*i*, *j*), which can be calculated as follows:

9 $$ FuncKL\left(i,j\right)=\frac{\sum_{d_t\in DS(i)}S\left({d}_t, DS(j)\right)+{\sum}_{d_t\in DS(j)}S\left({d}_t, DS(i)\right)}{\mid DS(i)\mid +\mid DS(i)\mid } $$

Here, |*D*(*i*)| and |*D*(*j*)| represent the number of diseases in *DS*(*i*) and *DS*(*j*) respectively. Thereafter, according to above Equation (9), it is obvious that a *N*_*L*_ × *N*_*L*_ dimensional lncRNA functional similarity matrix *FuncKL* can be obtained in final.

### Gaussian interaction profile kernel similarity of lncRNAs

Based on the assumption that similar lncRNAs tend to be more likely associated with similar diseases, according to above newly constructed lncRNA-disease association adjacency matrix *A*, for any two given lncRNAs *l*_*i*_ and *l*_*j*_, the Gaussian interaction profile kernel similarity between them can be obtained as follows:

10 $$ FKL\left({l}_i,{l}_j\right)=\mathit{\exp}\left(-{\gamma}_l{\left\Vert IP\left({l}_i\right)- IP\left({l}_j\right)\right\Vert}^2\right) $$

11 $$ {\gamma}_l={\gamma}_l^{\hbox{'}}/\left({\sum \limits}_{k=1}^{N_L}{\left\Vert IP\left({l}_k\right)\right\Vert}^2\right) $$

Here, *IP*(*l*_*t*_) denotes the vector consisting of elements in the *t*th column of the lncRNA-disease adjacency matrix *A*. *γ*_*l*_ is the parameter to control the kernel bandwidth based on the new bandwidth parameter${\gamma}_l^{\prime }$by computing the average number of lncRNAs-disease associations for all the lncRNAs. So far, based on above Equation (10), we can obtain a *N*_*L*_ × *N*_*L*_ dimensional lncRNA Gaussian interaction profile kernel similarity matrix *FKL* as well.

### Integrated similarity of lncRNAs

Based on the lncRNA functional similarity and lncRNA Gaussian interaction profile kernel similarity obtained above, a *N*_*L*_ × *N*_*L*_ dimensional integrated lncRNA similarity adjacency matrix *KL (N*_*L*_ *× N*_*L*_*)* can be obtained as follows:

12 $$ KL\left(i,j\right)=\frac{FuncKL\left(i,j\right)+ FKL\left(i,j\right)}{2} $$

### Construction of computational model TCSRWRLD

#### The establishment of heterogeneous network

Through combing the *N*_*D*_ × *N*_*D*_ dimensional integrated disease similarity adjacency matrix *KD* and the *N*_*L*_ × *N*_*L*_ dimensional integrated lncRNA similarity adjacency matrix *KL* with the *N*_*D*_ × *N*_*L*_ dimensional lncRNA-disease association adjacency matrix *A*, we can construct a new (*N*_*L*_ + *N*_*D*_) × (*N*_*L*_ + *N*_*D*_) dimensional integrated matrix *AA* as follow:

13 $$ AA\left(i,j\right)=\left[\begin{array}{cc} KL\left(i,j\right)& {A}^T\left(i,j\right)\\ {}A\left(i,j\right)& KD\left(i,j\right)\end{array}\right] $$

According to above Equation (13), we can construct a corresponding heterogeneous lncRNA-disease network consisting of *N*_*D*_ different disease nodes and *N*_*L*_ different lncRNA nodes, in which, for any given pair of nodes *i* and *j*, there is an edge existing between them, if and only if there is *AA*(*i*, *j*) > 0.

#### Establishment of TCS (target convergence set)

**Fig. 6:**
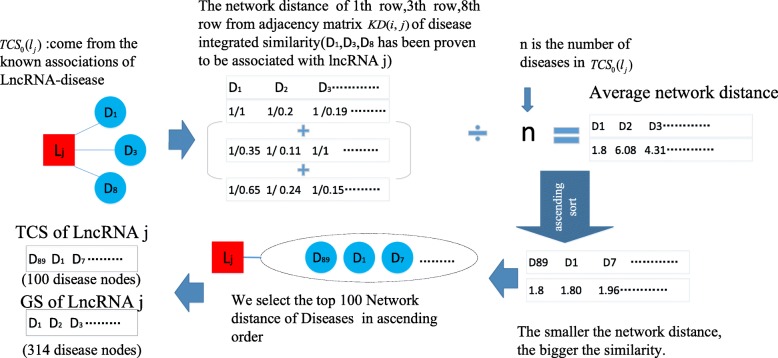
Flow chart of constructing TCS for an lncRNA node *j*

Before the implementation of random walk, for each node in above newly constructed heterogeneous lncRNA-disease network, as illustrated in Fig. 6, it will establish its own TCS first according to the following steps:

##### Step 1

For any given lncRNA node *l*_*j*_, we define its original TCS as the set of all disease nodes that have known associations with it, i.e., the original TCS of *l*_*j*_ is *TCS*_0_(*l*_*j*_) = {*d*_*k*_ | *A*(*k*, *j*) = 1, *k*∈[1, *N*_*D*_]}. Similarly, for a given disease node *d*_*i*_, we can define its original *TCS* as *TCS*_0_(*d*_*i*_) = {*l*_*k*_ | *A*(*i*, *k*) = 1, *k*∈[1, *N*_*L*_]}.

##### Step 2

After the original TCS has been established, for any given lncRNA node *l*_*j*_, ∀*d*_*k*_∈*TCS*_0_(*l*_*j*_), and ∀*t*∈[1, *N*_*D*_], then we can define the network distance *ND*(*k*, *t*) between *d*_*k*_ and *d*_*t*_ as follows:

14 $$ ND\left(k,t\right)=\frac{1}{KD\left(k,t\right)} $$

According to above Equation (14), for any disease nodes *d*_*k*_∈*TCS*_0_(*l*_*j*_) and ∀*t*∈[1, *N*_*D*_], obviously it is reasonable to deduce that the smaller the value of *ND*(*k*, *t*), the higher the similarity between *d*_*t*_ and *d*_*k*_ would be, i.e., the higher the possibility that there is potential association between *d*_*t*_ and *l*_*j*_ will be.

Similarly, for any given disease node *d*_*i*_, ∀*l*_*k*_∈*TCS*_0_(*d*_*i*_) and ∀*t*∈[1, *N*_*L*_], we can define the network distance *ND*(*k*, *t*) between *l*_*k*_ and *l*_*t*_ as follows:

15 $$ ND\left(k,t\right)=\frac{1}{KL\left(k,t\right)} $$

According to above Equation (15), for any lncRNA nodes *l*_*k*_∈*TCS*_0_(*d*_*i*_) and ∀*t*∈[1, *N*_*L*_], obviously it is reasonable to deduce that the smaller the value of *ND*(*k*, *t*), the higher the similarity between *l*_*t*_ and *l*_*k*_ will be, i.e., the higher the possibility that there is potential association between *l*_*t*_ and *d*_*i*_ will be.

##### Step 3

According to above Equation (14) and Equation (15), for any given disease node *d*_*i*_ or any given lncRNA node *l*_*j*_, we define that the TCS of *d*_*i*_ as the set of top 100 lncRNA nodes in the heterogeneous lncRNA-disease network that have minimum average network distance to the lncRNA nodes in *TCS*_0_(*d*_*i*_), and the TCS of *l*_*j*_ as the set of top 100 disease nodes in the heterogeneous lncRNA-disease network that have minimum average network distance to the disease nodes in *TCS*_0_(*l*_*j*_). Then, it is easy to know that these 100 lncRNA nodes in *TCS* (*d*_*i*_) may belong to *TCS*_0_(*d*_*i*_) or may not belong to *TCS*_0_(*d*_*i*_), and these 100 disease nodess in *TCS* (*l*_*j*_) may belong to *TCS*_0_(*l*_*j*_) or may not belong to *TCS*_0_(*l*_*j*_).

#### Random walk in the heterogeneous LncRNA-disease network

**Fig. 7:**
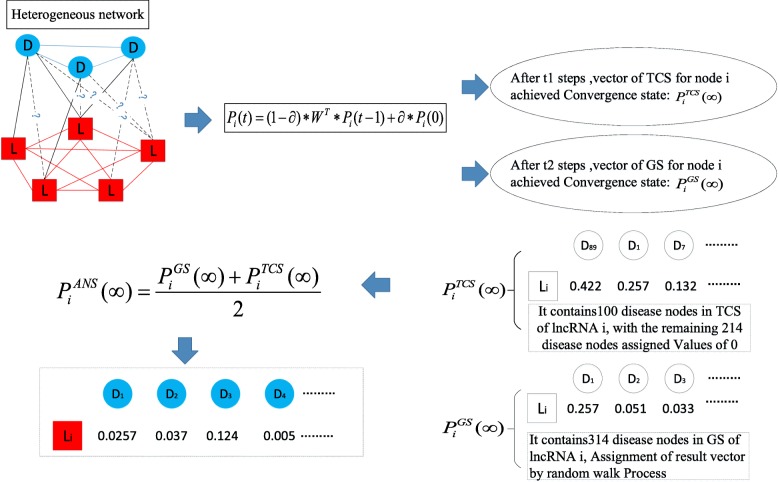
Flow chart of our prediction model TCSRWRLD

The method of random walk simulates the process of random walker’s transition from one starting node to other neighboring nodes in the network with given probability. Based on the assumption that similar diseases tend to be more likely associated with similar lncRNAs, as illustrated in Fig. 7, the process of our prediction model TCSRWRLD can be divided into the following major steps:

##### Step 1

For a walker, before it starts its random walk across the heterogeneous lncRNA-disease network, it will first construct a transition probability matrix *W* as follows:

16 $$ W\left(i,j\right)=\frac{AA\left(i,j\right)}{\sum_{k=1}^{N_D+{N}_L} AA\left(i,k\right)} $$

##### Step 2

In addition, for any node *£*_*i*_ in the heterogeneous lncRNA-disease network, whether *£*_*i*_ is a lncRNA node *l*_*i*_ or a disease node *d*_*i*_, it can obtain an initial probability vector *P*_*i*_ (0) for itself as follows:

17 $$ {P}_i(0)={\left({p}_{i,1}(0),{p}_{i,2}(0),\dots, {p}_{i,j}(0),\dots {p}_{i,{N}_D+{N}_L}(0)\right)}^T $$

18 $$ {p}_{i,j}(0)=W\left(i,j\right)\kern0.36em j=1,2,\dots, {N}_{D+}{N}_L $$

##### Step 3

Next, the walker will randomly select a node *§*_*i*_ in the heterogeneous lncRNA-disease network as the starting node to initiate its random walk, where *§*_*i*_ may be an lncRNA node *l*_*i*_ or a disease node *d*_*i*_. After the initiation of the random walk process, supposing that currently the walker has arrived at the node *Γ*_*i*_ from the previous hop node *Γ*_*j*_ after *t*-1 hops during its random walk across the heterogeneous lncRNA-disease network, then here and now, whether *Γ*_*i*_ is a lncRNA node *l*_*i*_ or a disease node *d*_*i*_, and *Γ*_*j*_ is a lncRNA node *l*_*j*_ or a disease node *d*_*j*_, the walker can further obtain a walking probability vector *P*_*i*_(*t*) as follows:

19 $$ {P}_i(t)=\left(1-\partial \right)\ast {W}^T\ast {P}_j\left(t-1\right)+\partial \ast {P}_i(0) $$

Where *∂* (0< *∂*< 1) is a parameter for the walker to adjust the value of walking probability vector at each hop. Moreover, based on above newly obtained walking probability vector *P*_*i*_(*t*), let *P*_*i*_(*t*) =${\left({p}_{i,1}(t),{p}_{i,2}(t),\dots, {p}_{i,j}(t),\dots {p}_{i,{N}_D+{N}_L}(t)\right)}^T$, and for convenience, supposing that there is *p*_*i*, *k*_(*k*)=maximum{${p}_{i,1}(t),{p}_{i,2}(t),\dots, {p}_{i,k}(t),\dots {p}_{i,{N}_D+{N}_L}(t)$}, then the walker will choose the node *ψ*_*k*_ as its next hop node, where *ψ*_*k*_ may be a lncRNA node *l*_*k*_ or a disease node *d*_*k*_. Especially, as for the starting node *§*_*i*_, since it can be regarded that the walker has arrived at *§*_*i*_ from *§*_*i*_ after 0 hops, then it is obvious that at the starting node *§*_*i*_, the walker will obtain two kinds of probability vectors such as the initial probability vector *P*_*i*_ (0) and the walking probability vector *P*_*i*_ (1). However, at each intermediate node *Γ*_*i*_, the walker will obtain two other kinds of probability vectors such as the initial probability vector *P*_*i*_ (0) and the walking probability vector *P*_*i*_(*t*).

##### Step 4

Based on above Equation (19), supposing that currently the walker has arrived at the node *Γ*_*i*_ from the previous hop node *Γ*_*j*_ after *t*-1 hops during its random walk across the heterogeneous lncRNA-disease network, let the walking probability vectors obtained by the walker at the node *Γ*_*i*_ and *Γ*_*j*_ be *P*_*i*_(*t*) and *P*_*j*_(*t*-1) respectively, if the L1 norm between *P*_*i*_(*t*) and *P*_*j*_(*t*-1) satisfies ‖*P*_*i*_(*t*) − *P*_*j*_(*t* − 1)‖_1_ ≤ 10^−6^, then we will regard that the walking probability vector *P*_*i*_(*t*) has reached a stable state at the node *Γ*_*i*_. Thus, after the walking probability vectors obtained by the walker at every disease node and lncRNA node in the heterogeneous lncRNA-disease network have reached stable state, and for convenience, let these stable walking probability vectors be ${P}_1\left(\infty \right),{P}_2\left(\infty \right),\dots, {P}_{N_D+{N}_L}\left(\infty \right)$, then based on these stable walking probability vectors, we can obtain a stable walking probability matrix *S*(∞) as follows:

20 $$ S\left(\infty \right)=\left[\frac{S_1}{S_3}\kern1em \frac{S_2}{S_4}\right]={\left({P}_1\left(\infty \right),{P}_2\left(\infty \right),\dots, {P}_{N_D+{N}_L}\left(\infty \right)\right)}^T $$

Where *S*_1_ is a *N*_*L*_×*N*_*L*_ dimensional matrix, *S*_2_ is a *N*_*L*_×*N*_*D*_ dimensional matrix, *S*_3_ is a *N*_*D*_×*N*_*L*_ dimensional matrix, and *S*_4_ is a *N*_*D*_×*N*_*D*_ dimensional matrix. And moreover, from above descriptions, it is easy to infer that the matrix *S*_2_ and the matrix *S*_3_ are the final result matrices needed by us, and we can predict potential lncRNA-disease associations based on the scores given in these two final result matrices.

According to above described steps of the random walk process based on our prediction model TCSRWRLD, it is obvious that for each node *Γ*_*i*_ in the heterogeneous lncRNA-disease network, the stable walking probability vector obtained by the walker at *Γ*_*i*_ is *P*_*i*_(∞) = ${\left({p}_{i,1}\left(\infty \right),{p}_{i,2}\left(\infty \right),\dots, {p}_{i,j}\left(\infty \right),\dots {p}_{i,{N}_D+{N}_L}\left(\infty \right)\right)}^T$. Moreover, for convenience, we denote a node set consisting of all the *N*_*D*_+*N*_*L*_ nodes in the heterogeneous lncRNA-disease network as a Global Set (*GS*), then it is obvious that we can rewrite the stable walking probability vector *P*_*i*_(∞) as ${P}_i^{GS}\left(\infty \right)$. Additionally, from observing the stable walking probability vector ${P}_i^{GS}\left(\infty \right)$, it is easy to know that the walker will not stop its random walk until the *N*_*D*_+*N*_*L*_ dimensional walking probability vector at each node in the heterogeneous lncRNA-disease network has reached a stable state, which will obviously be very time-consuming while the value of *N*_*D*_+*N*_*L*_ is large to a certain extent. Hence, in order to decrease the execution time and quicken the velocity of convergence of TCSRWRLD, based on the concept of TCS proposed in above section, while constructing the walking probability vector *P*_*i*_(*t*)=(*p*_*i*, 1_(*t*), *p*_*i*, 2_(*t*), …, *p*_*i*, *j*_(*t*), $\dots, {p}_{i,{N}_D+{N}_L}(t)\Big){}^T$ at the node *Γ*_*i*_, we will keep the *p*_*i*, *j*_(*t*) unchanged if the *j*th node in these *N*_*D*_+*N*_*L*_ nodes belongs to the TCS of *Γ*_*i*_, otherwise we will set *p*_*i*, *j*_(*t*)=0. Thus, the walking probability vector obtained by the walker at *Γ*_*i*_ will turn to be ${P}_i^{TCS}(t)$ while the stable walking probability vector obtained by the walker at *Γ*_*i*_ will turn to be ${P}_i^{TCS}\left(\infty \right)$. Obviously, comapred with ${P}_i^{GS}\left(\infty \right)$, the stable state of ${P}_i^{TCS}\left(\infty \right)$ can be reached by the walker much more quickly. However, considering that there may be nodes that are not in the TCS of *Γ*_*i*_ but actually associated with the target node, therefore, in order to avoid omissions, during simulation, we will construct a novel stable walking probability vector ${P}_i^{ANS}\left(\infty \right)$ through combining ${P}_i^{GS}\left(\infty \right)$with ${P}_i^{TCS}\left(\infty \right)$to predict potential lncRNA-disease associations as follows:

21 $$ {P}_i^{ANS}\left(\infty \right)=\frac{\ {P}_i^{GS}\left(\infty \right)+{P}_i^{TCS}\left(\infty \right)}{2} $$

## Supplementary information

**Additional file 1.** The known lncRNA-disease associations for constructing the known lncRNA-disease network. We list 1695 known lncRNA-disease associations which were collected from LncRNAdisease datasetit is the latest version in the database.

**Additional file 2.** The known 828 lncRNAs name Included in the 1695 known lncRNA-disease associations which were collected from LncRNAdisease datasetit is the latest version in the database.

**Additional file 3.** The known 314 diseases name Included in the 1695 known lncRNA-disease associations which were collected from LncRNAdisease datasetit is the latest version in the database.

**Additional file 4.** The known 98 human cancer,668 lncRNAs and 1103 confirmed associations between them from Lnc2Cancer database.

## Data Availability

The datasets generated and/or analysed during the current study are available in the LncRNADisease repository, http://www.cuilab.cn/ lncrnadisease.

## Abbreviations

10-Fold CV: 10-fold cross-validation
2-Fold CV: 2-fold cross-validation;
5-Fold CV: 5-fold cross-validation
AUC: Areas under ROC curve
AUPR: Area under the precision-recall curve
FPR: False positive rates
GS: Global set
H19: Long non-coding RNA H19
lncRNAs: Long non-coding RNAs
LOOCV: Leave-One Out Cross Validation
ncRNAs: Non-coding RNAs
P-R curve: Precision-recall curve
ROC: Receiver-operating characteristics
RWR: Random walk with restart
TCS: Target Convergence Set
TCSRWRLD: A novel computational model based on improved rand walk with restart is proposed to infer potential lncRNA-disease associations
TPR: True positive rates
Xist: Long non-coding RNA Xist
